# LEAD: From Devastation Comes Hope

**DOI:** 10.1289/ehp.118-a334

**Published:** 2010-08

**Authors:** Carol Potera

**Affiliations:** **Carol Potera**, based in Montana, has written for *EHP* since 1996. She also writes for *Microbe*, *Genetic Engineering News*, and the *American Journal of Nursing*

Even as the Gulf Coast grapples with possibly its worst environmental catastrophe ever, a silver lining has emerged from the devastation of the stormy summer of 2005: both soil lead levels and children’s blood lead levels fell dramatically across New Orleans, Louisiana, after Hurricanes Katrina and Rita swept in clean sediment over the city’s lead-contaminated soil.^1^ This positive outcome bolsters the case for soil remediation as a way to protect children from lead poisoning.

Howard Mielke, a research professor at the Tulane/Xavier Center for Bioenvironmental Research, says he and his colleagues “took advantage of a catastrophic natural event to examine changes in the environment and health.” Along with Sammy Zahran at Colorado State University and colleagues, Mielke measured soil lead levels at 46 New Orleans sites in 2000 and in 2006. The researchers obtained pre- and post-hurricane blood lead data for 13,306 children aged 6 years and younger from the Louisiana Childhood Lead Poisoning Prevention Program.

After the hurricanes, only 6 of the 46 sites had soil lead concentrations above 400 mg/kg, compared with 15 of 46 sites before the hurricanes. The 400 mg/kg (ppm) cutoff is the point at which the U.S. Environmental Protection Agency recommends remediation of bare soil in children’s play areas; a cutoff of 1,200 ppm is recommended for other bare soil areas. Median soil lead levels fell 46% (from 328.54 mg/kg to 203.33 mg/kg), and median blood lead declined 33% (from 5.14 μg/dL to 3.45 μg/dL). In neighborhoods where soil lead declined by 50% or more, blood lead dropped by 53% on average. Children born after Katrina and Rita had the lowest blood lead levels of all those studied.^1^

“There’s a tremendous amount of lead in New Orleans’ soil—and all cities,” says Mielke. This toxic reservoir, which accumulated when lead was added to paint and gasoline, is constantly being redistributed by rain, wind, and construction activity. The hurricanes’ blanket of cleaner soil—sediment from Lake Pontchartrain and nearby wetlands that was carried through the city’s breached levees by the storm surge—likely will not persist, predicts Mielke. However, the natural effect of the blanket of sediment is duplicated by soil remediation, in which clean soil with no more than 5 ppm lead is hauled in and deposited on a geotextile barrier. The barrier allows water to pass through but contains the lead and prevents anyone from digging into contaminated soil underneath.

Soil is often an underappreciated source of childhood lead exposure in cities, relative to lead paint in homes,^2^ yet “both are to blame for childhood blood lead elevation,” says Rudolph Jaeger, a research professor of environmental medicine at New York University Medical School. Exposure to lead in soil contributes to elevated blood lead, which in turn is associated with reduced educational outcomes.^3^

Mielke has worked with New Orleans area child-care centers where soil has contained 500–5,000 mg/kg lead. “If we pay attention to environments where children play in the very early years of life, we may reduce their blood lead levels,” Mielke says.

Mielke also thinks the current blood lead level of concern of 10 μg/dL—the level at which the Centers for Disease Control and Prevention recommends medical intervention—is too high. Studies show that just 2 μg/dL adversely impacts the heart,^4^ kidney,^5^ and child intelligence,^6^ and many researchers believe there is no safe level of exposure.^7^ “If we lower this threshold, there may be more interest in primary prevention measures like soil remediation,” Mielke says.

## Figures and Tables

**Figure:**
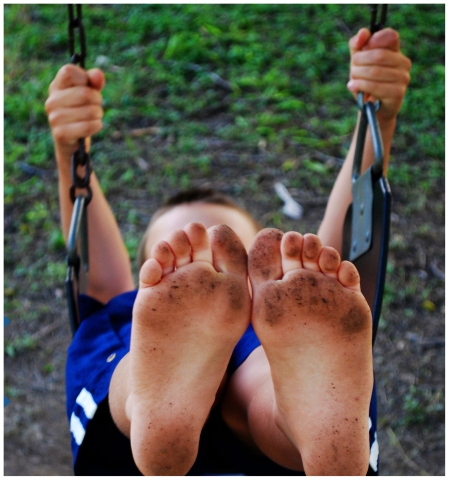
400 ppm Soil lead concentration at which the EPA recommends remediation of bare soil in children’s play areas 1,200 ppm Soil lead concentration at which the EPA recommends remediation of bare soil in other home areas
